# Vaccination Rates and Family Barriers Among Children with Inflammatory Bowel Disease

**DOI:** 10.1093/crocol/otaa056

**Published:** 2020-07-07

**Authors:** Kathleen J Holland, Tracey A Wilkinson, Erin Phipps, James E Slaven, William E Bennett

**Affiliations:** 1 Department of Pediatrics, Indiana University School of Medicine, Indianapolis, Indiana, USA; 2 Department of Pediatrics, Indiana University School of Medicine, Children’s Health Services Research, Indianapolis, Indiana, USA; 3 Department of Pediatrics, Indiana University School of Medicine, Developmental Pediatrics and Pediatric Gastroenterology, Hepatology and Nutrition, Indianapolis, Indiana, USA; 4 Department of Biostatistics, Indiana University School of Medicine, Indianapolis, Indiana, USA; 5 Department of Pediatrics, Pediatric Gastroenterology, Hepatology and Nutrition and Adolescent Comparative Effectiveness Research, Indiana University School of Medicine, Indianapolis, Indiana, USA

**Keywords:** children, inflammatory bowel disease, immunosuppression, vaccination, rates

## Abstract

**Background:**

Many children with inflammatory bowel disease (IBD) are taking immunosuppressant medications that place them at risk for vaccine preventable diseases. Despite national guidelines, children with IBD have low vaccination rates. Adult data suggest that there is concern about the safety of vaccines. There are no current studies addressing perceived safety about vaccinations among families of children with IBD.

**Methods:**

A total of 108 caregivers of children (ages 10–25 years) were surveyed during their outpatient visit, with approximately half having a diagnosis of IBD. The survey consisted of validated questions regarding vaccine safety and opinions. After enrollment, state-wide vaccine registry data was collected. Demographics between the 2 groups were compared using chi-square and the Wilcoxon rank-sum tests to analyze Likert-scale questions.

**Results:**

The majority of children followed for IBD were Caucasian males, had Crohn disease (68%), and were immunosuppressed. Results from the survey revealed a concern about vaccine safety (40% vs 16%, *P* = 0.03) and overall effectiveness (34% vs 12%, *P* < 0.01) in the IBD group compared with the non-IBD. Furthermore, more IBD families were worried that vaccines would worsen their child’s symptoms (36% vs 10%, *P* ≤ 0.01). The majority of children were missing the flu and/or human papilloma virus vaccine. Finally, 96% of the children on a biologic for their IBD were missing the PPSV23 booster.

**Conclusions:**

Caregivers of children with IBD are more concerned about vaccine safety and effectiveness than those with non-IBD diagnosis. Despite being on immunosuppressant medications, many patients were missing recommended vaccines.

## INTRODUCTION

The incidence of inflammatory bowel diseases (IBDs), which includes Crohn disease and ulcerative colitis, is increasing in incidence in the pediatric population and currently occurs at a rate of 10 per 100,000 children.^1^ The mainstays of treatment for IBD in children are immunosuppressant medications, including steroids, antimetabolites, and biologics.^2^ Immunosuppression places children at risk for vaccine preventable diseases.^3–7^ Due to this risk, the Centers for Disease Control and Prevention (CDC), the Infectious Disease Society of America (IDSA), the North American Society for Pediatric Gastroenterology, Hepatology, and Nutrition (NASPGHAN), and the American Academy of Pediatrics (AAP) have published guidelines for vaccination in this at-risk population.^8–10^

All immunosuppressed children should receive regularly scheduled childhood vaccinations (minus all live vaccines) with some additional vaccines to provide protection due to their immunosuppressed status.^8^ These recommendations include receiving yearly inactivated influenza, a pneumococcal booster with PPSV23, and the human papilloma virus (HPV) vaccine series.^8^ NASPGHAN also encourages practitioners to check hepatitis B virus (HBV), measles, mumps, and rubella (MMR), and varicella serologies, if feasible and when evidence of immunity is unknown and prior to initiating anti-tumor necrosis factor and provide booster immunizations if no serological response is present.^6, 7, 9^

Despite national vaccine guidelines, children and adults with IBD still have low rates of appropriate vaccination,^11–15^ and even when serologies are checked, booster vaccines are rarely given.^16, 17^ One small retrospective study assessed vaccination records for children with IBD starting biologic therapy and found only 67% had documentation of a complete primary vaccination series.^16^

Barriers to and beliefs about vaccinations have been studied in adults but have not been assessed in families of children with IBD who are immunosuppressed. Adult studies show they are concerned not only about vaccine safety and effectiveness but also feel that there is a general lack of communication between primary care providers and subspecialists. Adult patients believe it is the responsibility of both parties to discuss vaccination safety.^12, 18^

Pediatric subspecialists have the advantage of following more medically complex patients frequently; however, studies have demonstrated that they do not always use these opportunities to address vaccinations, despite NASPGHAN encouraging pediatric gastroenterologists to coordinate care and counsel about vaccines in concordance with the primary care physician.^9^ Surveys find that most subspecialists agree that vaccinations should be handled by the primary care provider (PCP), but almost 50% believed that some education should be provided in the subspecialist’s office.^19^ With adequate training of gastroenterologists around vaccination counseling, studies prove that rates can improve, even in a subspecialty setting.^20–22^

All previous studies in the pediatric IBD population have focused on clinicians’ attitudes toward vaccinations, and to our knowledge, there has been no study examining parental perspectives. We sought to compare current vaccination rates and parental beliefs toward vaccine safety in patients both with and without IBD followed in a tertiary care children’s hospital.

## MATERIALS AND METHODS

### Population

Families with children ages 10–25 years were recruited between March and October 2019 during a routine office visit in the exam or infusion rooms at a free-standing children’s hospital in Indianapolis, IN. IBD patients were approached during their follow-up visit for IBD or during their biologic infusion. Control patients were approached during their general gastroenterology follow-up visit. We had no specific exclusion criteria. Demographic information was collected for the child and parent including: age, sex, ethnicity, race, insurance type, highest educational level of the caregiver, employment of the parent or patient, and dual-parent versus single-parent household. We also abstracted clinical data from the patient’s chart including type of IBD (Crohn disease, ulcerative colitis, or indeterminate colitis), current IBD medications, and history of gastrointestinal (GI)-related surgeries as well as primary GI diagnosis for those patients without IBD.

### Survey

A survey regarding parental attitudes toward vaccines was provided to one of the primary caregivers via an online secure electronic database.^23, 24^ The survey was completed in the exam room by the parents, while consent was obtained via 2 trained research assistants just prior to each scheduled appointment. The questions were derived from a 2011 study by Opel et al that used questions from previous surveys to identify “vaccine-hesitant parents.” ^25^ A 4-point Likert scale was used. Families of children with IBD were asked disease-specific questions regarding severity and compared with families of children without IBD, who are followed for a different GI disorder. Families were compensated for their time.

### Vaccination Records

We determined the rate of vaccination for immunizations recommended for immunosuppressed children with IBD and those that require checking serologies prior to initiating biologic therapy: HPV, influenza (2018–2019 season), PCV7/PCV13 and PCV23, MCV, MMR, varicella, and hepatitis B. Indiana utilizes an online registry, the Children and Hoosier Immunization Program (CHIRP), which was used by research assistants to access vaccination records for each participant. All clinics, including primary care and subspecialty offices across the state, are mandated to upload vaccination records within 7 days of receiving a vaccine into the CHIRP registry. This mandate was set in 2015. The only vaccine available at the pediatric gastroenterology office is an inactivated influenza vaccine. All other vaccines are provided by PCPs.

### Statistical Analysis

Demographics and question responses were analyzed to see whether there was significant heterogeneity between groups using chi-square tests, which are presented in the tables. Fisher exact tests were also performed when expected cell counts were small to ensure these were similar to the chi-square tests. Due to the ordinal nature of the Likert-scale questions (questions 8–17), these were analyzed using Wilcoxon rank-sum tests. Medians between the IBD and non-IBD groups were also calculated using the ranking system: Strongly Agree = 4, Agree = 3, Unsure = 2, and Disagree = 1. All analytic assumptions were verified, and analyses were performed using SAS v9.4 (SAS Institute, Cary, NC).

## RESULTS

A total of 108 families were recruited for the study, 50 of which had a child being followed for IBD and another 58 served as a comparison group who had a child with a non-IBD GI complaint. Demographics for the parent and child are shown in Table 1. Most of the children followed for IBD were males (56% compared with 41%) and mostly white (79% non-IBD vs 80% in the IBD group) with a mean age of 15 years old for the IBD group and 14 for the non-IBD group (median 16 years for IBD and 15 for non-IBD). The only statistically significant difference between the 2 groups was a larger proportion of Hispanic children among the IBD group (16% vs 3%, *P* = 0.03) as well as Hispanic parents (14% vs 0%, *P* < 0.01) More of the parents of children with IBD had extended school beyond college when compared with controls (52% vs 14% and *p* ≤ 0.01) (Table 1). Fourteen percent of parents of the IBD cohort changed their opinion regarding vaccines after their child was diagnosed with IBD compared with 3% of the non-IBD parents (*P* = 0.06).

**TABLE 1.** Child/Parent Demographics and Disease Characteristics

	IBD Group	Non-IBD Group	
	n = 50 (%)	n = 58 (%)	*P*
Parent demographics			
Gender			
Male	6 (17)	7 (12)	0.46
Female	44 (88)	51 (88)	
Age (median)	45 (SD 6.42)	44.5 (SD 8.37)	0.731
Number of children (median)	2.7 (SD 1.05)	2.7 (SD 1.17)	1.0
Race			
White	44 (88)	52 (90)	0.741
Black	4 (8)	5 (9)	0.854
Other	2 (4)	1 (2)	0.541
Ethnicity			
Non-Hispanic	43 (86)	58 (100)	0.003
Hispanic	7 (14)	0 (0)	
Level of education			
Less than high school	4 (8)	13 (22)	0.05
Some college	20 (40)	37 (64)	0.013
Graduate school	26 (52)	8 (14)	<0.001
Currently employed	37 (74)	44 (76)	0.812
Single parent	14 (28)	14 (24)	0.637
Annual household income			
<25,000	6 (12)	9 (16)	0.122
25–75,000	17 (34)	25 (43)	0.341
>75,000	27 (54)	24 (41)	0.179
Child demographics			
Gender			
Male	28 (56)	24 (41)	0.122
Female	22 (44)	34 (59)	
Age (median)	15 (SD 2.2)	14 (SD 2.92)	0.050
Race			
White	40 (80)	46 (79)	0.898
Black	4 (8)	4 (7)	0.844
Other	6 (12)	8 (14)	0.760
Ethnicity			
Non-Hispanic	42 (84)	56 (97)	0.025
Hispanic	8 (16)	2 (3)	
Seen PMD in last year	48 (96%)	53 (91%)	0.47
Parent reported vaccines up-to-date	47 (94%)	57 (98%)	0.33
Delayed vaccines due to something other than illness	8 (16%)	6 (10%)	0.41
Vaccine opinion changed following GI diagnosis (parent response)	7 (14%)	2 (3%)	0.06
Disease characteristics			
Crohn	34 (68)	N/A	
Ulcerative colitis	15 (30)	N/A	
Indeterminate	1 (2)	N/A	
Current medications		N/A	
Anti-TNF (infliximab/ adalimumab)	48 (96)	N/A	
Immunomodulator^a^	14 (28)	N/A	
Steroids	4 (8)	N/A	
Mesalamine	1 (2)	N/A	
History of abdominal surgeries^b^	7 (14)		
GI symptoms			
Abdominal pain	N/A	33 (57)	
Acid reflux	N/A	18 (31)	
Constipation	N/A	17 (29)	
Vomiting	N/A	14 (24)	
IBS	N/A	14 (24)	
Diarrhea	N/A	12 (21)	
Liver/gallbladder	N/A	10 (17)	
Pancreatitis	N/A	1 (2)	
Other	N/A	16 (28)	

IBS = irritable bowel syndrome; N/A = not applicable; PMD = primary medical doctor; TNF = tumor necrosis factor.

^a^Immunomodulator includes azathioprine, methotrexate or 6-mercaptopurine.

^b^Abdominal surgeries include abscess drainage, resections and/or dilations.

Disease characteristics are also available in Table 1. The majority of children in the IBD group had Crohn disease (68%) and more than 90% were on the biologic infliximab (92%). A small percentage of children were on a steroid (8%), and only 7% of all IBD patients had a history of a GI-related surgery. Among the non-IBD group, 57% were followed for abdominal pain, 31% for acid reflux, and 29% for constipation (Table 1).

Results from the parent survey are shown in Table 2. Significant differences between the IBD and non-IBD group were noted. The non-IBD group felt stronger that they trusted information on vaccines provided by their doctors (median score 4 vs 3; *P* = 0.007), that they could openly discuss vaccines with their PCP (median 4 vs 3.5; *P* = 0.001), and strongly believed vaccines prevent serious illness (median 4 vs 3; *P* = 0.011). Most of the non-IBD group strongly disagreed with the statement that children receive too many vaccines compared to the IBD group (median 1 vs 2; *P* = 0.007). More of the non-IBD group believed that it is the responsibility of the PCP to discuss vaccines (median 4 vs 3; *P* = 0.011).

**TABLE 2.** Family Opinion Regarding Vaccines

	IBD				Non-IBD Group				
General	Strongly Agree	Agree	Unsure	Disagree	Strongly Agree	Agree	Unsure	Disagree	*P*
Trusts information from physicians regarding vaccines	21 (42)	22 (44)	7 (14)	0 (0)	39 (67)	16 (28)	3 (5)	0 (0)	0.007
Comfortable discussing concerns with PMD	25 (50)	24 (48)	1 (2)	0 (0)	47 (81)	11 (19)	0 (0)	0 (0)	0.001
Believe vaccines prevent serious illness	21 (42)	26 (52)	2 (4)	1 (2)	40 (69)	14 (24)	4 (7)	0 (0)	0.011
Believe children get more vaccines than are necessary for them	1 (2)	11 (22)	17 (34)	20 (40)	3 (5)	3 (5)	13 (22)	39 (67)	0.007
Pediatric GI specialists should discuss vaccines	7 (14)	30 (60)	5 (10)	7 (14)	8 (14)	24 (41)	13 (22)	13 (22)	0.105
PMD should discuss vaccines	24 (48)	25 (50)	0 (0)	1 (2)	42 (72)	15 (26)	1 (2)	0 (0)	0.011
Vaccine Concerns	Very Concerned	Concerned	Unsure	Not Concerned	Very Concerned	Concerned	Unsure	Not Concerned	
Level of concern about vaccine safety	7 (14)	20 (40)	6 (12)	17 (34)	10 (17)	9 (16)	8 (14)	31 (53)	0.084
Vaccines could worsen child’s GI illness	2 (4)	18 (36)	16 (32)	14 (28)	4 (7)	6 (10)	9 (16)	39 (67)	<0.001
Vaccines may not be effective due to child’s GI illness	2 (4)	17 (34)	15 (30)	16 (32)	3 (5)	7 (12)	8 (14)	40 (69)	0.001

PMD = XXX.

The majority of the differences significant in the IBD group were concerned about vaccine safety and effectiveness compared with the non-IBD group. Although nonsignificant, 40% of the IBD group were concerned about the safety of vaccines compared with 16% of non-IBD group (median 1 vs 3; *P* = 0.084), and 36% were concerned that vaccines might worsen their child’s IBD compared with 10% of the non-IBD group being concerned that vaccines would worsen their underlying GI illness (median 1 vs 2; p ≤ 0.001). Last, the IBD families were more concerned about the effectiveness of vaccines, with 34% compared with 12% (median 1 vs 2; *P* = 0.001).

Results from the vaccine registry are shown in Table 3. Registry data were available on 45 participants in the IBD group and 54 in the non-IBD group. Based on the available information, none of the patients on the biologic agents including infliximab or adalimumab had received a PPSV23. Fifty percent of the non-IBD and 51% of the IBD groups were missing at least one of the HPV vaccines in the series or had never received one. This was not statistically significant between the 2 groups. Forty percent of the IBD children and 40% of the non-IBD children had never been given one of the HPV vaccines in the series.

**TABLE 3.** Missing Vaccines Between Children with IBD Versus Non-IBD

	IBD Group	Non-IBD Group	
	n = 45 (%)	n = 54 (%)	*P*
HPV-9^a^	23 (51)	27 (50)	0.921
Influenza^b^	25 (56)	36 (67)	0.264
PCV7/PCV13^c^	40 (89)	48 (89)	1.0
PPSV23^d^	40 (89)	N/A	
MCV4^e^	4 (9)	7 (13)	0.531
MMR^f^	1 (2)	3 (6)	0.324
Varicella^g^	2 (4)	4 (7)	0.521
Hepatitis B^h^	0 (0)	6 (11)	0.022
Hepatitis A^i^	11 (24)	6 (11)	0.087
Tdap	3 (7)	6 (11)	0.494

^a^No vaccines or missing doses in the 2- or 3-dose series of HPV-9 vaccine.

^b^Missing an inactivated influenza vaccine the previous flu season.

^c^Missing at least one dose of PCV7/PCV13 to complete the schedule.

^d^Missing PPSV23.

^e^Missing any MCV4.

^f^Missing at least one of the 2 dose MMR vaccines.

^g^Missing at least one of the 2 dose varicella vaccines.

^h^Missing at least one of the 3 dose HBV vaccines.

^i^Missing at least one of the 2 dose series HAV vaccines.

Most children were missing an influenza vaccine from the 2018 to 2019 season with 56% of those in the IBD group compared with 67% among the non-IBD group (*P* = 0.264). Six of the children in the non-IBD group were missing at least one of the HBV vaccines, whereas all of the IBD group had completed the series (*P* = 0.022). Fifty-one percent of the children with IBD were missing any or at least one of the HPV-9 vaccines in the series compared with 50% among the non-IBD group (*P* = 0.921). Among this group, 40% of the IBD and 40% of the non-IBD had never received any HPV-9 vaccine. Ninety-six percent of the children with IBD in our cohort were on a biologic agent and 8% were on steroids. Among this group, 89% were missing a PPSV23 vaccine. The majority of children in both groups were up to date on the MMR and varicella (Table 3).

## DISCUSSION

We performed a cross-sectional study of vaccine attitudes in the families of children with and without IBD in the pediatric gastroenterology office. Our results show that important differences exist between these 2 groups and that pediatric gastroenterologists are in a unique position to improve the rates of essential vaccines in this high-risk population.

Vaccine rates for special vaccine considerations were low in this studied IBD population and despite guidelines and expert opinion within the field. Additional vaccine considerations for children with chronic inflammatory conditions including autoimmune diseases who are immunosuppressed is set forth by the Infectious Disease Society of America with more recent evidence placed on the importance of the HPV vaccine series.^3, 8^ This study shows that the majority of patients with IBD are missing a seasonal influenza vaccine along with any/one of the HPV vaccines and PPSV23 (unique to those who are immunosuppressed). Eleven of 45 participants with IBD (24%) had received 1 HPV vaccine in the series but had not received a second vaccine. The non-IBD group were also frequently missing an influenza vaccine and/or an HPV vaccine in the series similar to the IBD group indicating that this is not necessarily an issue only seen in an immunosuppressed population but in all children.

HPV vaccination has increased slightly from 65.5% to 68.1% from 2017 to 2018 but remains below national goals.^26^ Among 18,700 adolescents surveyed in 2019, 51% were fully vaccinated compared with 48.6% in 2017 and the proportion that received at least one dose increased from 65.5% to 68.1%. Overall, girls are being vaccinated against HPV at higher rates compared to boys (53.7% girls compared to 48.7% boys).^27^ Our study falls in a similar pattern. Most of the children in the IBD group were males, which may have changed the results slightly given that a larger proportion of children in the non-IBD group were female. All studies in adult IBD groups have focused on females, but males are also at risk for throat, penile, and anus cancer, and males often act as silent carriers of the virus.^28^

Low influenza vaccination rates have been analyzed in previous studies. Influenza vaccination is frequently presented as an “opt-in” vaccine rather than the “opt-out” methodology, which may explain some of the low rates.^29^ National data from the CDC are encouraging in that the percentage of children vaccinated against the flu has increased from 51.5% to 62.6% between 2017 and 2019 seasons, but again, this remains lower than the national goal.^30^

Results of the parent survey bring to light several important points, some of which may explain lower vaccination rates. One is that more of the IBD population are concerned about the safety of vaccines, concerned that vaccines will worsen their child’s IBD and that vaccines will be less effective given their child’s IBD. This is novel information in the pediatric IBD population, but we can see that the fear of vaccine safety did not change the overall vaccination rates when compared to non-IBD groups. Perhaps even above the concern about safety is the general misunderstanding about vaccines including influenza, HPV and PPSV23. A larger percentage of parents in the IBD group changed their opinion about vaccines after their child was diagnosed with IBD compared with the parents of the non-IBD control group (14% vs 3%). The question was not worded in a way to understand whether the change in opinion was a positive or negative one and thus hard to interpret with the final vaccination rates.

Overall, families of children with IBD and those without believe pediatric gastroenterologists should discuss vaccines along with the PCPs. Previous studies have established specific barriers among subspecialists including time constraints, needing additional education about vaccines and communication barriers with PCPs offices.^19^ Important to also note that >90% of families (both IBD and non-IBD) believe that their child is “up-to-date” when in fact they are missing vaccines. Perhaps families are unaware of what “up-to-date” means given that the most frequently missed vaccines are those not always mandated by schools.

Limitations to this study include that it was performed at a single tertiary care center where attitudes and physician practice may be relatively homogenous and the sample size was small. Enrollment was based on convenience sampling. This is evident in the fact that most of the children with IBD were those receiving infliximab. Infliximab infusions may take up to 3 hours to complete and make it easier for completion of consent and parent survey. Additionally, we did not assess a healthy control population as our comparator, but rather non-IBD patients followed in the GI clinic, since our aim was to help provide clinical guidance to gastroenterologists caring for these 2 groups. This choice might present a bias given that all children for this study were followed at a tertiary children’s hospital and could be medically complex. Fifty-two percent of IBD parents had attained some graduate education and 88% of parents were white. This may partially explain the lower vaccination rates in the IBD group. Previous research has shown lower vaccination rates (particularly the HPV vaccine) in children of parents who are white and those with a higher level of education.^31^

Another important limitation is the use of CHIRP registry data. Given that CHIRP has been mandated as of 2015 in Indiana, it is possible some of the early childhood vaccinations, including hepatitis B (missing in 6 of the non-IBD groups) were not updated in the registry. Influenza, PPSV23 and HPV should all be updated within the registry based on the age of our cohorts and time since the CHIRP mandate. The HPV vaccine series can be offered up until age 26, and with the majority of the IBD cohort being 15 years, there are still several years for this vaccine to be given.

## CONCLUSION

This study highlights barriers identified in families of children with IBD and shows that despite expert opinion, certain important vaccines for immunosuppressed children are missed. Families again believe that both the subspecialists and the PCP should address vaccines in clinic. Future studies may need to take this information to practitioners to provide families and providers the education and tools needed to discuss vaccines in clinic with the hopes of improving vaccination rates.

## Notes

Funding: Funding for this project was awarded by the Department of Pediatrics Grant for Trainees in the amount of $10,000 (grant number UL1TR002529).

Data Availability: Data stored and supported on figshare.com at https://doi.org/10.6084/m9.figshare.12442757.v1.
